# Investigation of Eating Patterns Among Individuals With Chronic Urticaria

**DOI:** 10.7759/cureus.101453

**Published:** 2026-01-13

**Authors:** Merve Akay, Elif Ozulku, Merve Kaya, Burak Celik, Gülhan Aksoy Saraç

**Affiliations:** 1 Dermatology, Ankara Bilkent City Hospital, Ankara, TUR; 2 Clinical Research, Başkent University Faculty of Medicine, Ankara, TUR; 3 Dermatology, Cook County Health, Chicago, USA

**Keywords:** chronic spontaneous urticaria, eating behaviour, eating disorders (eds), emotional eating, food-related exacerbation, pseudoallergen-free diet, quality-of-life, uncontrolled eating, urticaria, urticaria activity score

## Abstract

Chronic urticaria (CU) is a long-lasting inflammatory skin disease, characterized by recurrent wheals and/or angioedema lasting more than six weeks, often impairing quality of life. Recent findings suggest that eating behavior may play a role in CU symptom exacerbation. This study aimed to evaluate eating behaviors in patients with chronic spontaneous urticaria (CSU) and explore their relationship with disease activity and duration. A total of 83 CSU patients and 81 age- and sex-matched healthy controls were included in the study. Participants completed the Three-Factor Eating Questionnaire-Revised 18 (TFEQ-R18) to assess eating behaviors, and Beck Depression (BDI) and Anxiety (BAI) Inventories to evaluate psychological symptoms. Disease activity was measured using the Urticaria Activity Score over seven days (UAS7). Statistical analyses were performed using IBM SPSS Statistics for Windows, Version 26 (Released 2019; IBM Corp., Armonk, New York, United States), with p<0.05 considered significant. Patients with CSU had significantly higher BDI (11.11 ± 7.65 vs. 5.14 ± 4.26; p<0.001) and BAI (12.76 ± 9.32 vs. 5.02 ± 5.91; p<0.001) scores compared to controls. Uncontrolled eating scores were significantly higher in the CSU group (46.54 ± 19.88 vs. 34.06 ± 9.22; p<0.001), whereas emotional eating and cognitive restraint did not differ significantly. UAS7 scores showed a strong positive correlation with uncontrolled eating (r=0.515, p<0.001) and a moderate correlation with emotional eating (r=0.376, p<0.001). Patients with CSU exhibit significantly higher uncontrolled eating behaviors, which are positively associated with disease activity, independent of depression or anxiety. Addressing disordered eating patterns may contribute to improved disease management and quality of life in patients with CU.

## Introduction

Chronic urticaria (CU) is a common inflammatory dermatological condition, characterized by the recurrence of itchy wheals and/or angioedema lasting more than six weeks, leading to a marked impairment in patients’ quality of life. According to current guidelines, CU can be classified as chronic inducible urticaria (CIndU) and chronic spontaneous urticaria (CSU) [1].

CIndU is defined by clear, subtype-specific triggers that are reproducible, wheals, angioedema, or both, which always occur when the trigger is present and never occur when it is absent. However, some patients with CSU may also experience trigger-related wheals and/or angioedema. These triggers are not absolute, as their presence does not always result in symptoms. In addition, wheals and/or angioedema can occur spontaneously, without any identifiable trigger [1,2]. Such triggers include physical or external stimuli such as cold, heat, ultraviolet light, vibration, water, pressure, and exercise.

In recent years, it has been hypothesized that eating may represent one of the inducible factors in CU. Recent studies have shown that eating can exacerbate symptomatic dermographism (SD), leading to the description of new subtypes such as food-dependent and food-exacerbated SD [3,4]. In this observational case-control study, we aim to investigate eating behaviors in patients with CU and their relationship with disease duration and activity. This study was previously presented as an abstract at the 2025 European Academy of Dermatology and Venereology (EADV) Symposium on May 24, 2025 at Prague, Czech Republic.

## Materials and methods

This prospective study included 85 age- and sex-matched patients with CSU and 85 healthy controls aged 18-65 years. CSU patients under a minimum of six months’ follow-up in our outpatient clinic, and those compliant with a pseudoallergen-low diet were included in the study. Patients with known endocrinologic, rheumatologic, chronic liver, kidney, neurological, oncological, or psychiatric diseases, as well as obese patients and pregnant individuals, were excluded from the study.

Urticaria Activity Score over 7 days (UAS7) scores and disease duration were collected from the patients [5,6]. Additionally, Depression- and anxiety-related symptoms were evaluated with the Beck Depression Inventory (BDI) and Beck Anxiety Inventory (BAI) [7-11]. Patients and controls completed the Three-Factor Eating Questionnaire-Revised 18 (TFEQ-R18) for the evaluation of eating behaviors [12-14].

UAS7 is a validated patient-reported outcome measure that assesses disease activity in chronic urticaria by recording the severity of pruritus (Weekly Itch Severity Score) and the number of wheals (Weekly Hives Severity Score) over seven days. Each component is scored from 0 to 21, resulting in a total UAS7 score ranging from 0 to 42. Lower scores indicate milder symptoms (UAS7=0 signifies no itch or wheals), while higher scores reflect more severe disease activity (UAS7=42 represents maximal itch and wheals) [5,6].

The Beck Depression Inventory (BDI) is a self-rated scale consisting of 21 items designed to assess the severity of depression. Each item is scored from 0 to 3, with higher total scores indicating greater levels of depression [7,8]. The Beck Anxiety Inventory (BAI) is a similar self-reported scale used to evaluate anxiety levels. Similar to the BDI, it consists of 21 questions, each scored from 0 to 3, about how the individual has felt in the preceding week regarding common anxiety symptoms such as sweating, tremor, fear, and feelings of distress [9]. The validity and reliability study of the Turkish version of the inventory, originally developed by Beck et al., was conducted by Hisli and Ulusoy et al., respectively [10,11].

The Three-Factor Eating Questionnaire-Revised 18 (TFEQ-R18) is a revised version of the original TFEQ, first developed by Karlsson et al., and consists of 18 items on a 4-point Likert scale (definitely true, mostly true, mostly false, definitely false). Responses to each of the 18 items are given a score from 1 to 4. The TFEQ-R18 corresponds to three aspects of eating behavior: cognitive restraint, uncontrolled eating, and emotional eating [12,13]. The Turkish validity and reliability of the questionnaire were studied by Kirac et al. [14]. The raw scale scores are transformed to a 0-100 scale ((raw score - lowest possible raw score)/possible raw score range × 100). Higher values on the respective scales are more indicative of the aforementioned behaviors.

The IBM SPSS Statistics for Windows, Version 26 (Released 2019; IBM Corp., Armonk, New York, United States) was used for the statistical analysis of data. The Shapiro-Wilk test was used for the evaluation of distribution. Continuous data with a normal distribution were presented as mean ± standard deviation and tested using the Student’s t-test; otherwise, they were analyzed using the Mann-Whitney U test. Categorical data were expressed as number and percentage. Spearman or Pearson correlation analyses were used for the evaluation of correlation, as appropriate. A p-value<0.05 was considered statistically significant. The required sample size was determined using the G*Power (Ver. 3.1 Heinrich-Heine-Universität Düsseldorf, Düsseldorf, Germany) software. An a priori power analysis for an independent samples t-test was conducted with a significance level of α=0.05, statistical power (1-β)=0.90, and an expected effect size of Cohen’s d=0.5. The analysis indicated that approximately 84 participants were required in each group.

## Results

A total of 83 patients and 81 controls completed the study. Two patients and four participants from the control group did not complete the questionnaire and were therefore excluded from the analysis. The patient group consisted of 61 female subjects and 22 male subjects, while the control group included 62 female participants and 19 male participants. The mean age of the patient group was 38.61 ± 4.42 years, and the mean age of the control group was 38.1 ± 3.82 years, with no significant difference between the groups (p>0.05). The mean BMI was 24.81 ± 2.68 in the patient group and 24.1 ± 3.13 in the control group, with no significant difference (p=0.12). The mean disease duration in the patient group was 7.02 ± 6.49 years, and the mean UAS7 score was 24.69 ± 8.78.

**Figure 1:**
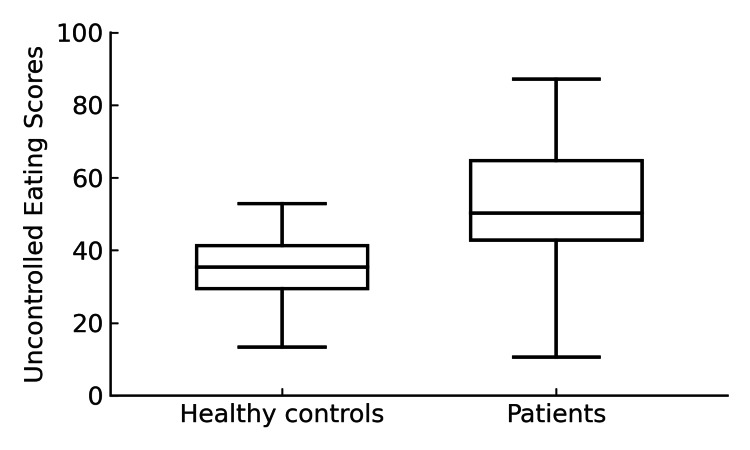
Comparison of the uncontrolled eating scores of the patient and control groups

The BDI score was significantly higher in the patient group (11.11 ± 7.65) compared to the control group (5.14 ± 4.26; p<0.001). Similarly, the BAI score was significantly higher in the patient group (12.76 ± 9.32) than in the control group (5.02 ± 5.91; p<0.001). Regarding TFEQ-18 scores, the uncontrolled eating score was significantly higher in the patient group (46.54 ± 19.88) than in the control group (34.06 ± 9.22; p<0.001) (Figure 1).

However, there was no significant difference between the groups in emotional eating and cognitive restraint eating scores (Table 1).

**Table 1:** Comparison of eating behavior, BDI and BAI scores between the patient and control groups BDI: Beck depression inventory, BAI: Beck anxiety inventory, SD: standard deviation.

	Patients (n=83)			Controls (n=81)			
	Mean	Median	SD	Mean	Median	SD	p-value
Emotional eating	35.2	33.33	26.36	34.84	33.4	20.3	0.758
Restrictive eating	48.52	50	15.32	45.26	44.44	14.54	0.127
Uncontrolled eating	46.54	44.45	19.88	34.06	33.34	9.22	0.001
BDI	11.11	9	7.65	5.14	4	4.26	<0.001
BAI	12.76	9.12	9.32	5.02	3	5.91	<0.001

When analyzing the correlations between UAS7 scores and depression, anxiety, and TFEQ-18 subscales, a strong positive correlation was found between UAS7 and uncontrolled eating (r=0.515, p<0.001). Additionally, there was a moderate positive correlation between UAS7 and emotional eating (r=0.376, p<0.001). However, there was no significant correlation between UAS7 scores and anxiety or depression scores (p=0.302 and p=0.972, respectively) (Table 2).

**Table 2:** Correlation of UAS7 scores with the BDI and BAI scores of the eating subgroup BDI: Beck depression inventory, BAI: Beck anxiety inventory, UAS7: Urticaria activity score-7.

	UAS7	
	p-value	rho
Emotional eating	<0.001	0.376
Restrictive eating	0.08	0.399
Uncontrolled eating	<0.001	0.515
BDI	0.72	0.401
BAI	0.302	0.115

In the correlation analysis between BDI scores and TFEQ-18 subscales, a weak positive correlation was observed only between cognitive restraint eating and BDI scores (rho=0.281, p=0.01), while no significant correlation was found for uncontrolled eating or emotional eating. Finally, no significant correlations were found between disease duration and depression, anxiety, or TFEQ-18 scores (p>0.05) (Table 3).

**Table 3:** Correlation of disease duration with the BDI and BAI scores of the eating subgroup BDI: Beck depression inventory, BAI: Beck anxiety inventory.

	Disease duration	
	p-value	rho
Emotional eating	0.051	-0.225
Restrictive eating	0.684	0.045
Uncontrolled eating	0.605	0.058
BDI	0.755	0.035
BAI	0.587	0.060

## Discussion

CU negatively impacts quality of life, and the presence of psychiatric comorbidities such as depression, anxiety, or somatoform disorders further exacerbates this effect in affected patients [15]. Similar to our study, Engin et al. showed higher depression and anxiety scores in patients with CU compared to healthy controls using the BDI and BAI [16]. Other studies have also reported an increased prevalence of psychiatric comorbidities, including depression and anxiety, in patients with CU, suggesting a potential psychosomatic association in the disease pathophysiology. These findings were not correlated with disease activity or duration, similar to previous studies [15,16]. This could indicate that psychological distress in CU may not be solely driven by symptom severity but could instead be related to the unpredictable nature of the disease, its chronic course, and its impact on daily life.

Our study showed that patients have higher uncontrolled eating behavior compared to healthy controls, regardless of their depression or anxiety levels. The relationship between uncontrolled eating and UAS7 showed a positive correlation in our study, suggesting that disordered eating patterns may contribute to increased disease activity in CU. In previous studies, Latzer et al. described a patient with uncontrolled urticaria and angioedema induced by binge/purge eating episodes [17]. The higher frequency of uncontrolled eating episodes may have led to higher rates of disease activity in patients with urticaria. Similar to our study, Ertas et al. showed that eating may exacerbate symptomatic dermographism among patients with CInU [4]. Their findings, in line with ours, suggest that dietary factors and eating behaviors may play a role in urticaria symptom severity and disease course. Emerging evidence suggests that low-grade immune activation in the gut may contribute to systemic inflammation, potentially amplifying mast-cell-mediated pathways involved in the pathogenesis of urticaria. Furthermore, disturbances in the gut-brain axis may enhance neuroimmune stress responses, providing a plausible link between psychological factors and the exacerbation of urticaria symptoms [18]. Additionally, uncontrolled eating has various negative effects on individuals’ lives. It has been linked to reduced executive and cognitive functioning [19]. Yeomans et al. showed that people with higher uncontrolled eating scores are more prone to impulsiveness and risk-taking [20].

Our study has several limitations. The primary limitation is its cross-sectional design, which prevents us from determining whether these findings are the cause or consequence of urticaria. Although UAS7 was used to assess disease activity, a validated measure of disease control, such as the Urticaria Control Test (UCT), was not included; therefore, the potential relationship between psychological parameters or eating behaviors and overall disease control could not be evaluated. The lack of a disease-control instrument also limits interpretation of whether BDI and BAI scores may influence long-term treatment response or symptom stability rather than activity alone. Moreover, data on individual food triggers and dietary patterns were not collected, which prevents us from assessing how specific food-related factors may interact with uncontrolled eating behaviors or contribute to disease exacerbation. Collecting monthly diet and food-consumption records together with simultaneously documented urticaria activity and disease-control scores in future studies may provide greater insight into the effect of uncontrolled eating on disease outcomes. Therefore, larger longitudinal or interventional studies are needed to better explore the potential contribution of uncontrolled eating episodes in triggering or worsening urticaria.

Another limitation of our study is that patients on a pseudoallergen-low diet were included. However, due to the cross-sectional nature of our study, we could not completely determine patients’ compliance with their diet [21]. Due to our study’s methodology, BMI-matched patients were selected, and the impact of uncontrolled eating on weight gain or metabolic syndrome could not be clearly analyzed.

In conclusion, the current study demonstrated that patients with CU exhibit higher levels of uncontrolled eating behavior compared to healthy controls, regardless of their depression or anxiety levels. Additionally, we found a strong positive correlation between uncontrolled eating and disease activity, suggesting that disordered eating patterns may contribute to symptom severity in urticaria. Assessing and addressing uncontrolled eating patterns in patients with CU could provide a new approach to symptom management.

## Conclusions

In this study, we demonstrated that patients with CSU exhibit significantly higher levels of uncontrolled eating behavior compared with healthy controls, independent of their depression or anxiety levels. Furthermore, uncontrolled eating was strongly associated with higher UAS7 scores, suggesting that disordered eating patterns may contribute to increased disease activity in chronic urticaria. Emotional eating also showed a moderate positive correlation with disease activity, supporting the emerging evidence that behavioral and neuroimmune factors may play a role in the exacerbation of urticaria symptoms.

These findings highlight the importance of evaluating eating behaviors as a potential component of disease management in chronic urticaria. Incorporating dietary assessment, screening for disordered eating patterns, and providing appropriate behavioral guidance may help optimize symptom control and improve quality of life in affected individuals. Future studies that include disease-control measures and detailed dietary data are needed to further clarify the relationship between eating behaviors, psychological factors, and long-term urticaria outcomes.
